# Supplementary material for: Germacrene A Synthases for Sesquiterpene Lactone Biosynthesis Are Expressed in Vascular Parenchyma Cells Neighboring Laticifers in Lettuce
Source: Plants (Basel). 2022 Apr 28;11(9):1192. doi: 10.3390/plants11091192 (PMC9099558; doi:10.3390/plants11091192)
Supplement: Supplementary file 1 [file plants-11-01192-s001.zip › plants-1631991-supplementary.pdf]

Supplementary data

Table S1. The list of primers used in this study.

No.	Sequences
1	CCGAGTGGACGAACAGTGGTT
2	ACCCATACCCACTAAGGCAGATT
3	CAAAAGCCATGGAAGCCCCAAA
4	AAACCAAGACGGTGGACGGAATA
5	TCCTTGGGGCTTAACCAGTCCCA
6	TCAGCCCAGATGGAAGGTGGGAA
7	GGAGATGAGGCACAATCCAAAAGAGG
8	CACGGAGCTCGTTGTAGAAAGTGTGA
9	**AAAAAGCAGGCT**TCGGGGGTCCTTAACCTTATGAACG
10	**AGAAAGCTGGGT**CTTCTTGAAGTTGAAAGGGTATAGAAAAGC
11	**AAAAAGCAGGCT**TCGAGAGAATATAATCAAACAAAATCGAACC
12	**AGAAAGCTGGGT**CTTCTTGAAATTGAATTGGGGTTTG
13	GGGGACAAGTTTGTACA**AAAAAGCAGGCT**
14	GGGGACCACTTTGTACA**AGAAAGCTGGGT**

Note: Overlapping sequences in 9/11/13 and 10/12/14 are shown in bold.
